# Involvement of TGFBI-TAGLN axis in cancer stem cell property of head and neck squamous cell carcinoma

**DOI:** 10.1038/s41598-024-57478-0

**Published:** 2024-03-21

**Authors:** Motoharu Sarubo, Yasuhiro Mouri, Akira Moromizato, Azusa Yamada, Shengjan Jin, Wenhua Shao, Hiroko Hagita, Keiko Miyoshi, Yasusei Kudo

**Affiliations:** Department of Oral Bioscience, Tokushima University Graduate School of Biomedical Sciences, Tokushima, Japan

**Keywords:** TGFBI, Partial-EMT, Transgelin, Cancer stem cell, Head and neck squamous cell carcinoma, Oral cancer, Cancer stem cells

## Abstract

Head and neck squamous cell carcinoma (HNSCC) is a significant healthcare burden globally. Previous research using single-cell transcriptome analysis identified TGFBI as a crucial marker for the partial-epithelial-mesenchymal transition (partial-EMT) program. However, the precise role of TGFBI in HNSCC progression remains unclear. Therefore, our study aimed to clarify the impact of TGFBI on the malignant behavior of HNSCC cells. Through RNA-sequencing data from the TCGA database, we validated that increased TGFBI expression correlates with a higher occurrence of lymph node metastasis and unfavorable prognosis in HNSCC cases. Functional experiments demonstrated that TGFBI overexpression enhances the ability of sphere formation, indicating stem-cell-like properties. Conversely, TGFBI depletion reduces sphere formation and suppresses the expression of cancer stem cell (CSC) markers. RNA-sequencing analysis of TGFBI-overexpressing and control HNSCC cells revealed TAGLN as a downstream effector mediating TGFBI-induced sphere formation. Remarkably, TAGLN depletion abolished TGFBI-induced sphere formation, while its overexpression rescued the suppressed sphere formation caused by TGFBI depletion. Moreover, elevated *TAGLN* expression showed correlations with the expression of *TGFBI* and partial-EMT-related genes in HNSCC cases. In conclusion, our findings suggest that TGFBI may promote CSC properties through the upregulation of TAGLN. These novel insights shed light on the involvement of the TGFBI-TAGLN axis in HNSCC progression and hold implications for the development of targeted therapies.

## Introduction

Head and neck squamous cell carcinoma (HNSCC) is a widespread malignancy affecting various organs such as the oral cavity, pharynx, and larynx. Globally, HNSCC accounts for over 500,000 cases annually, with a 5-year survival rate of approximately 50%^1^. Unfortunately, despite advances in therapeutic approaches, survival rates have remained stagnant for more than two decades^2^. The primary risk factors for HNSCC are smoking and alcohol consumption. Like other cancers, HNSCC undergoes a stepwise progression characterized by the accumulation of genetic and epigenetic alterations^3^. During this progression, cancer cells transition from an epithelial to a mesenchymal phenotype through a process known as Epithelial-to-Mesenchymal Transition (EMT)^4,5^. Recently, the concept of partial-EMT has emerged, representing an intermediate state during EMT induction^6^. Notably, cancer cells in the partial-EMT state exhibit enhanced migration, metastatic potential, and resistant to treatment, indicating its contribution to malignant behavior^7^. Consequently, the ability of cancer cells to undergo partial-EMT, rather than complete EMT, is associated with a higher risk of metastasis. Through single cell transcriptome analysis, Puram et al*.* classified HNSCC into five subtypes: “Epithelial differentiation,” “Cycling,” “Stress,” “Hypoxia,” and “partial-EMT”^8^. Remarkably, the “partial-EMT” subtype demonstrates the closest association with metastasis and poor prognosis. Moreover, HNSCC cells exhibiting partial-EMT characteristics are localized at the leading edge of tumors, in close proximity to the surrounding stroma^8^. Thus, partial-EMT represents a pivotal event in HNSCC progression.

*TGFBI*, a gene responsive to transforming growth factor beta (TGF-β) signaling, encodes a protein with distinctive structural features. The TGFBI protein encompasses a signal peptide sequence for secretion, an EMI domain rich in cysteine residues, four homologous fasciclin 1 (FAS1) domains at the N-terminus, and an arginine-glycine-aspartate (RGD) integrin binding motif at the C-terminus. Notably, TGFBI is implicated in the pathogenesis of corneal dystrophies, a group of progressive inherited corneal disorders^9^. Additionally, TGFBI plays significant roles in regulating diverse biological functions, including cell adhesion, embryonic development-related bone formation, and the pathogenesis of various human diseases^10^. In the context of cancer, TGFBI has been associated with both tumor suppressor and promotion. However, its role in tumor progression remains controversial and whether TGFBI contribute to malignant behaviors in HNSCC cells has yet to be elucidated. Hence, the aim of this study was to investigate the involvement of TGFBI in HNSCC progression.

## Materials and methods

### Reagent and antibodies

Antibodies were obtained from the following companies: Anti-human TGFBI polyclonal antibody (LS-C332168, LSBio, Shirley, MA, USA), Anti-human TAGLN antibody (6G6, sc-53932, Santa Cruz), Anti-HA-probe polyclonal antibody (Y-11, sc-805, Santa Cruz), and Anti-ß-actin monoclonal antibody (A-5441, SIGMA). FAK Inhibitor 14 was obtained from Santa Cruz. Puromycin and G418 were obtained from FUJIFILM Wako Pure Chemical Corp., Osaka, Japan.

### Cell culture

SAS, HSC3, SCC-4, OSC20, HSC4, OSC19, HSC2, KON, Ca9-22, SAT, and Ho-1-U-1 were obtained from JCRB (Japanese Collection of Research Bioresources Cell Bank). These cells were maintained in Dulbecco's Modified Eagle's Medium (FUJIFILM Wako) supplemented with heat-inactivated 10% fetal bovine serum (Nichirei Bioscience Inc., Tokyo, Japan) and 1% Penicillin and Streptomycin (FUJIFILM Wako) at 37 °C in 5% CO_2_. For growth assay, 5 × 10^3^ cells were plated onto 96-well plates, and measured cell proliferation by using Cell Counting Kit-8 (Dojindo, Kumamoto, Japan), according to the manufacturer’s instructions.

### Quantitative RT-PCR

By using the RNeasy Mini Kit (Qiagen), total RNA was isolated from cells. Their purity was determined by a standard spectrophotometric method. From total RNA, cDNA was synthesized by using the PrimeScript RT Master Mix (Takara Bio Inc). mRNA expression was determined by using a CFX connect real time system (Roche) with SYBR Premix Ex Taq II reagent (Takara Bio Inc). For quantitation of total *TGFBI, TAGLN, ALDH1, BCL11B* or *GAPDH* transcripts, the following primer sequences were used. *TGFBI*: forward, 5′-CATCAGGGCTCAACACGATG-3′ and reverse, 5′-TGTTCAGCAGGTCTCTCAGG-3′; *TAGLN*: forward, 5′-TCAAGCAGATGGAGGAGGTG-3′ and reverse, 5′-GCTGCCATGTCTTTGCCTTC-3′; *ALDH1*: forward, 5′-GTGTTGAGCGGGCTAAGAAG-3′ and reverse, 5′-CCAGTTTGGCCCCTTCTTTC-3′; *BCL11B*: 5′-CTCATCACCCAGGCTGACC-3′ and reverse, 5′-ACACTGCTTCCTTTTGTGCT-3′; *NANOG*: forward, 5′-CCTGTGATTTGTGGGCCTG-3′ and reverse, 5′-GACAGTCTCCGTGTGAGGCAT-3′; *POU5F1*: forward, 5′-GTGGAGGAAGCTGACAACAA-3′ and reverse, 5′-ATTCTCCAGGTTGCCTCTCA-3′; *SOX2*: forward, 5′-GTATCAGGAGTTGTCAAGGCAGAG-3′ and reverse, 5′-TCCTAGTCTTAAAGAGGCAGCAAAC-3′; *GAPDH*: forward, 5′-TCCACCACCCTGTTGCTGTA-3′ and reverse, 5′-GCATCCTGGGCTACACTGAG-3′. Relative mRNA expression of each transcript was normalized against *GAPDH* mRNA.

### Western Blot analysis

Western blotting was performed as described previously^11^. TGFBI-overexpressing Ho-1-U-1 and HSC3 cells were treated with 3 μM of monensin (Sigma, Burlington, USA) for 24 h. The intracellular protein expression of TGFBI is low because of the secretion from intracellular into extracellular. To quantify intracellular protein expression of TGFBI, we utilized monensin known as a protein transport inhibitor. Monensin blocks intracellular protein transport processes of any secreted proteins and induces the accumulation of these proteins in the Golgi complex. The increased accumulation of secreted proteins enhances the detectability of TGFBI with Western blot analysis utilizing total cell lysate. Cells were lysed using lysis buffer (50 mM pH 7.6 Tris–HCl, 150 mM NaCl, 1 mM EDTA, 1.5 mM MgCl2, 0.5% Nonidet P-40, and 10% glycerol) with protease inhibitor cocktail (Nacalai tesque, Kyoto, Japan). After centrifugation, the supernatant was collected. Protein concentration was measured using Thermo Fischer BSA protein assay reagent (Thermo Scientific) by the absorption at 562 nm using a microplate reader (TECAN infinite 200Pro). Using 5–20% gradient polyacrylamide gel (ATTO Corporation, Tokyo, Japan), these proteins were electrophoresed followed by blotting onto a nitrocellulose membrane (GE Healthcare Life Science). By using a Western ECL Substrate (BIO-RAD), the signal was detected by an Amersham ImageQuant 800 (GE Healthcare Life Science).

### Plasmids and transfection

For generating TGFBI-overexpressing cells, pcDNA3.1^+^-C-(k)DYK plasmid encoding full-length human TGFBI (GenScript) was transfected into HNSCC cells by using Fugene HD (promega), according to the manufacturer’s instructions. After transfections, we treated the cells with 500 µg/mL of G418, and then we picked up clones (clone and pool clone).

For TAGLN overexpression and knockdown of TGFBI, lentiviral vectors, pLV[shRNA]-EGFP:T2A:Puro-U6 (Scramble_shRNA and hTGFBI) and pLV[Exp]-mCherry/Neo-EF1A (hTAGLN) were obtained from Vector Builder. Lentiviral packaging plasmids (gag-pol, rev, VSVG cording plasmids 1: 1: 1 mix) were provided from Dr. Guardavaccaro (University of Verona). Lentiviral vectors and packaging plasmids were transfected into Lenti-X 293 T cells (Takara Bio Inc) by using TransIT-293 transfection reagent (Mirus Bio), according to the manufacturer’s instructions. Then, supernatants were collected at 48 h after transfection and filtered using 0.45 μm membrane. Filtered supernatants with 8 μg/ml polybrene directly infected to HNSCC cells. After 24 h, the medium was replaced by fresh media with 500 µg/mL G418 or 1 µg/mL Puromycin.

### RNA interference

Logarithmically growing cells were seeded at a density of 3 × 10^5^ cells/6-cm dish and transfected with oligonucleotides by using Oligofectamine RNAi MAX (Invitrogen), according to the manufacturer’s instructions. TGFBI siRNAs were obtained from Ambion Life Technologies Corporation Silencer® select Pre-designed (Inventoried) siRNA Product (s14070, #ASO2L6OI), as the following sequences: TGFBI, 5′-GCAUGACCCUCACCUCUAUtt -3′. After 2 days, we passaged these cells to the other dishes and cultured more 2 days. Then we used these cells for other experiments.

### Sphere formation assay

HNSCC cells were seeded into 6-well Ultra low attachment surface plates (Corning, 5 × 10^4^ cells/well) or 24-well Ultra low attachment surface plates (1 × 10^4^/well). After 4–7 days, the cells were cultured to allow the generation of spheroids. After generating spheroids, we counted the number of spheroids with a size of 100 µm or more, which is visible for naked eyes.

### In vitro invasion assay

In vitro invasion assay was described previously^11^. Invasiveness was determined by using a 24-well cell culture insert with 8 µm pores (#3097, Becton Dickinson). The filter was coated with 50 µg Matrigel (Becton Dickinson) as a reconstituted basement membrane substance. The lower compartment contained 0.5 mL of culture media. Following trypsinization, 1.5 × 10^5^ cells were plated on the upper compartment of the cell culture insert with 100 μL culture media. After 24 h incubation, the cells on the upper surface of the filter were removed by wiping with a cotton swab, and the cells on the lower surface of the filter were fixed with formalin and stained with hematoxylin (FUJIFILM Wako). The number of invaded cells (cells on the lower surface of the filter) was counted under a light microscope.

### Bulk RNA-seq analysis

mRNA was isolated from total RNA using the KAPA mRNA Capture kit (KAPA, cat. KK8440) and cDNA libraries were prepared using the MGIEasy RNA Directional Library Prep Set (MGI Tech, cat. 1,000,006,385). Sequencing was performed using DNBSEQ-G400RS with a 150-bp paired-end configuration. The quality check and adaptor removal were performed using *FastQC* and *fastp* programs on the raw read sequences. The paired-end reads were mapped to the human reference genome (GRCh38 primary assembly genome) using the *STAR* software. After mapping, read counts and TPM values were generated using the *RSEM* software. Raw RNA-seq data (FASTQ files) reported in this study are available at the DDBJ Sequence Read Archive (DRR486141-DRR486143).

### scRNA-seq analysis

The log-transformed gene expression values from single-cell RNA-seq data from HNSC (GSE103322) was analyzed using the Seurat (v4.3.0). As the quality control, genes expressed in less than 5 cells and cells with more than 10,000 genes were filtered. We performed dimensionality reduction using Uniform Manifold Approximation and Projection (UMAP) with the top 14 principal components. The resolution parameter was set to 0.4 for the FindClusters function. We determined the cell types of each cluster based on the marker genes identified using the FindAllMarkers function.

### Data analysis

Clinical and gene expression information of 514 head and neck squamous cell carcinoma (TCGA-HNSC) samples were obtained from the cBioPortal website (https://www.cbioportal.org/) for primary lymph node presentation assessment analysis, neoplasm histologic grade analysis, gene expression analysis, and survival analysis. The five-year overall survival of HNSC patients in the top 25% and bottom 25% expression groups for *TGFBI*, *TAGLN*, and *TGFBI*/*TAGLN* was analyzed using the *survival* and *survminer* R packages. The gene expressions (partial-EMT related genes, *NANOG*, *POU5F1*, *SOX2*, *ALDH1*, *BCL11B*, *S100A4*, *VIM*, *TGFBI*, and *TAGLN*) of HNSCC patients in the top 25% and bottom 25% expression groups for TGFBI, TAGLN, or TGFBI/TAGLN was analyzed on cBioPortal website. Also, the correlations of TGFBI expression with several gene expressions (FN1, LGALS3BP, MT1F, TAGLN, MME) in HNSCC patients were analyzed on cBioPortal website. The log-transformed TPM values of HNSC cell lines (DepMap 23Q2 files) were obtained from the depmap portal website (https://depmap.org/portal/). The scores of six gene programs (CellCycle, Epidif.1/2, Stress, Hypoxia, and pEMT) in each HNSC cell line were calculated using ssGSEA with the *ConsensusTME* R package.

### Statistical analysis

For Kaplan–Meier overall survival, the log rank test was used for comparing two groups. For sphere formation assay, the Welch’s t-test or ANOVA test was used for comparing groups. For in vitro invasion assay, chemoresistance assay, the Welch’s t-test was used for comparing groups. For quantitative RT-PCR analysis, the t-test was used for comparing to control value. For correlation of gene expression using TCGA database, the Student’s t-test was used for comparing two group.

## Results

### Correlation of *TGFBI* expression with partial-EMT phenotype in HNSCC cell lines

To assess the correlation between *TGFBI* expression, lymph node metastasis, and prognosis in HNSCC cases, we analyzed processed RNA-seq data from the TCGA database. Notably, high *TGFBI* expression exhibited a higher incidence of lymph node metastasis compared with low *TGFBI* expression (Supplementary Fig. S1A). Furthermore, HNSCC cases with high expression of *TGFBI* demonstrated a poor prognosis (Supplementary Fig. S1B). By using previous scRNA-seq data^8^, higher *TGFBI* expression was observed in cancer cells and cancer stromal fibroblasts (Supplementary Fig. S1C). Indeed, higher expression levels of TGFBI mRNA and protein was observed in HNSCC cell lines (Supplementary Fig. S2A,B). Among 11 HNSCC cell lines, HSC3, Ca9-22, and Ho-1-U-1 displayed lower expression levels. Previous study classified HNSCC into five subtypes: “Epithelial differentiation,” “Cycling,” “Stress,” “Hypoxia,” and “partial-EMT,” using single-cell transcriptome analysis^8^. By leveraging the gene expression profiles of 11 HNSCC cell lines, we assigned them to the respective subtypes (Supplementary Fig. S2C). Among the cells, SCC-4, OSC20, OSC19, and HSC2 were classified as the partial-EMT subtype. Although the expression levels of *TGFBI* did not always align with the partial-EMT program in HNSCC cells, HSC3, Ca9-22 and Ho-1-U-1, characterized by low *TGFBI* expression, exhibited a diminished partial-EMT phenotype.

### Enhanced sphere formation by TGFBI in HNSCC cells

**Figure 1:**
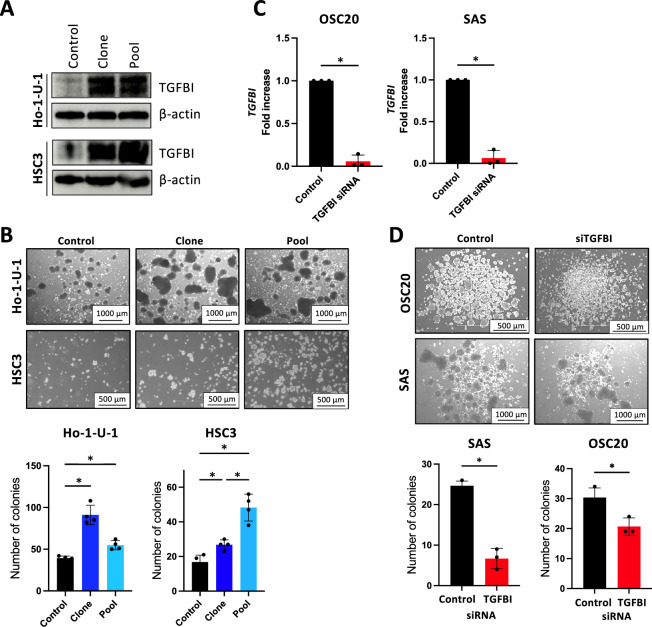
*Sphere formation by TGFBI in HNSCC cells.* (**A**) Immunoblotting confirming ectopic TGFBI expression in TGFBI-overexpressing Ho-1-U-1 and HSC3 cells (clone and pool cells for each cell line). β-actin served as the loading control. (**B**) Assessment of sphere formation using Ultra low attachment surface plates. Control, clone, and pool TGFBI-overexpressing Ho-1-U-1 and HSC3 cells were employed. 5 × 10^4^ cells were seeded into 6-well Ultra low attachment surface plates. Images of spheroid formation are shown after 4 days (Ho-1-U-1 cells) and 6 days (HSC3 cells) in upper panel. Lower panel shows the number of spheroids with a size of 100 µm or more after 4 days (Ho-1-U-1 cells) and 6 days (HSC3 cells). Data represent the mean ± SD in each group (n = 4). *P-value < 0.05. (**C**) Quantitative RT-PCR analysis of *TGFBI* expression in OSC20 and SAS cells transfected with TGFBI siRNA. *P-value < 0.05. (**D**) Assessment of sphere formation using Ultra low attachment surface plates. Control and TGFBI-knockdown OSC20 and SAS cells were used. 5 × 10^4^ cells were seeded into 6-well Ultra low attachment surface plates. Images of spheroid formation are shown after 6 days in upper panel. Lower panel shows that the number of spheroids with a size of 100 µm or more after 6 days. Data represent the mean ± SD of triplicates in each group. *P-value < 0.05.

To elucidate the role of TGFBI, we generated TGFBI-overexpressing cells using Ho-1-U-1 and HSC3 cells with low *TGFBI* expression (Fig. 1A). We obtained both TGFBI-overexpressing clone and pool clone of Ho-1-U-1 and HSC3 cells. Compared to control cells, TGFBI-overexpressing cells exhibit a sheet-like cell mass with a smooth margin shape (Supplementary Fig. S3A). While TGFBI overexpression suppressed cell proliferation and invasion (Supplementary Fig. S3B,C), it significantly enhanced sphere formation ability (Fig. 1B). Sphere-forming culture is well known for effectively enriching subpopulations with stem-cell properties. Indeed, TGFBI-overexpressing cells exhibited an increased number of colonies even when plated with a small number of cells (1000 cells/well and 500 cells/well) (Supplementary Fig. S4). To further demonstrate the phenotype induced by TGFBI overexpression, we utilized validated TGFBI siRNA in OSC20 and SAS cells with high *TGFBI* expression (Supplementary Figure S2A,B). Treatment with TGFBI siRNA effectively reduced *TGFBI* expression in both cell lines (Fig. 1C). Subsequently, we examined sphere formation in these cells and found that TGFBI depletion significantly decreased the number of colonies formed (Fig. 1D).

Thus, TGFBI was involved in sphere formation of HNSCC cells, indicating stem-cell-like properties. Indeed, SCC-4 cells with a partial-EMT phenotype showed higher expression of stem cell and CSC markers compared to other HNSCC cell lines (Supplementary Fig. S5A). Therefore, we investigated the expression of stemness markers, such as *NANOG*, *OCT4*, and *SOX2* following the overexpression of TGFBI (Ho-1-U-1 and HSC3 cells). Although TGFBI overexpression tended to elevate the expression levels of stemness markers, we did not observe a significant difference (Supplementary Fig. S5B). Moreover, we examined CSC markers, *ALDH1* and *BCL11B* in TGFBI-overexpressing cells (Ho-1-U-1 and HSC3) and TGFBI-depleted cells (OSC20 and SAS). TGFBI overexpression tended to elevate the expression levels of CSC markers and TGFBI depletion exhibited reduced expression of CSC markers (Supplementary Fig. S5C,D).

### Involvement of TAGLN in TGFBI-promoted sphere formation

**Figure 2:**
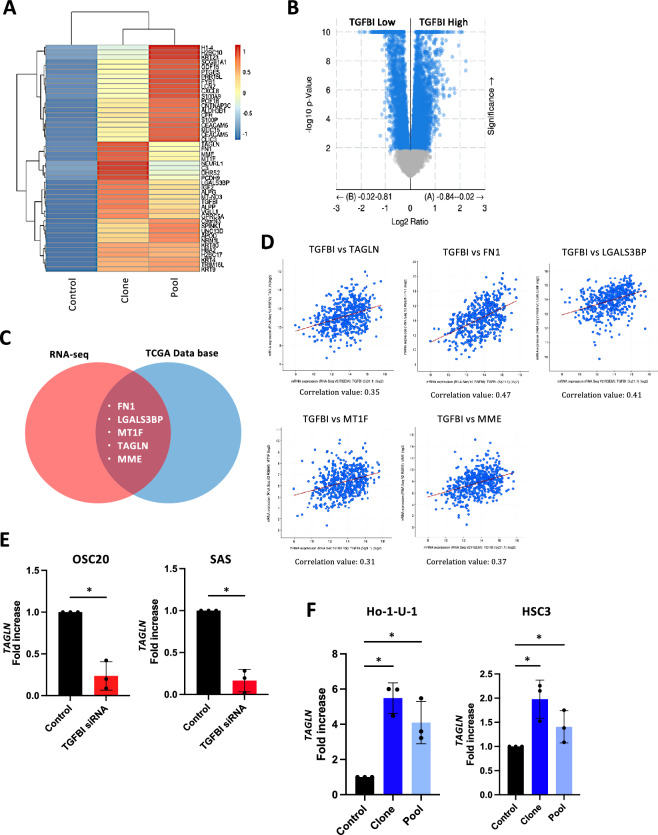
*Identification of TAGLN in TGFBI-mediated signaling.* (**A**) RNA-sequencing results of TGFBI-overexpressing Ho-1-U-1 cells (clone and pool) and control cells. Heat map displays the most related gene to *TGFBI* expression in Ho-1-U-1 cells. For generating heat map, we used these cut-off value, “Clone: log_2_(Clone expression/control expression) > 1” and “Pool: log_2_(Pool expression/ control expression) > 0.5”. (**B**) HNSCC patient gene expression profiles from TCGA Firehose Legacy. TCGA HNSCC patient gene expression analysis was used for realizing *TGFBI* related genes. (**C**) For identifying TGFBI-related genes by comparing RNA-sequencing results with HNSCC patient gene expression profiles from TCGA Firehose Legacy. Five genes (FN1, LGALS3BP, MT1F, TAGLN, and MME) were picked up by the following requirement, RNA-seq: log_2_(Clone expression/Pool expression) > 1, TCGA: Spearman’s rank correlation coefficient > 0.3 (*P*-value < 0.05), and Pearson’s correlation coefficient > 0.3 (*P*-value < 0.05). (**D**) Correlation of *TGFBI* expression to *TAGLN, FN1, LGALS3BP, MT1F, and MME* expression in HNSCC patients using data from TCGA Firehose Legacy. (**E**) Quantitative RT-PCR analysis of *TAGLN* expression in control and TGFBI-knockdown OSC20 and SAS cells. Data represent the mean ± SD of triplicates in each group. *P-value < 0.05. (**F**) Quantitative RT-PCR analysis of TAGLN expression in control, clone, and pool TGFBI-overexpressing Ho-1-U-1 and HSC3 cells. Data represent the mean ± SD of triplicates in each group. *P-value < 0.05.

To pursuit unraveling the molecular mechanisms behind TGFBI-mediated sphere formation, we compared gene expression profiles of TGFBI-overexpressing clone and pool Ho-1-U-1 cells with control Ho-1-U-1 cells by RNA-sequencing analysis (Fig. 2A). TGFBI overexpression resulted in the upregulation of genes involved in negative regulation of cell population proliferation, angiogenesis, response to oxidative stress, and others, while downregulating genes involved in the regulation of GTPase activity, Wnt signaling pathway, organelle assembly Ras signaling, and more (Supplementary Fig. S6). Among the upregulated genes in TGFBI-overexpressing cells, we checked the genes with high expression of *TGFBI* in HNSCC cases from the TCGA database (Fig. 2B,C). *FN1* (Fibronectin 1), *LGALS3BP* (galectin 3 binding protein), *MT1F* (metallothionein 1F), *TAGLN* (transgelin), and *MME* (membrane metalloendopeptidase) were identified as a gene with a strong correlation with TGFBI (Fig. 2C,D). Notably, only *TAGLN* was significantly downregulated by TGFBI depletion in HNSCC cells (Fig. 2E). Furthermore, we confirmed that *TAGLN* expression was upregulated by TGFBI-overexpressing cells (Fig. 2F). TAGLN depletion decreased *TGFBI* expression, that it may be caused by positive feedback (Supplementary Figure S7A).

**Figure 3:**
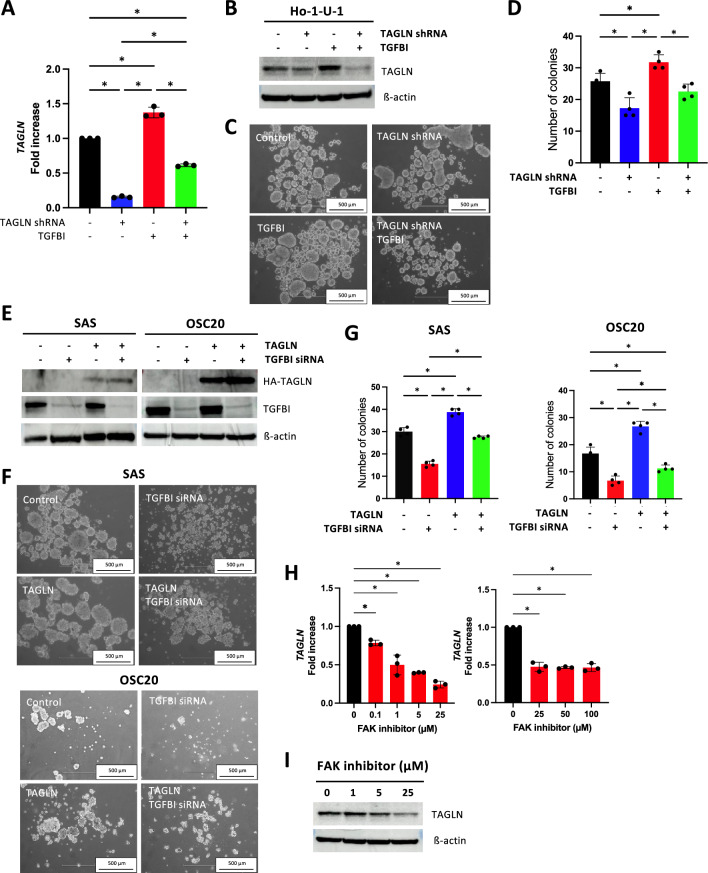
Involvement of TAGLN in TGFBI-promoted sphere formation. (**A**) Quantitative RT-PCR analysis of *TAGLN* expression in TAGLN shRNA-transfected TGFBI-overexpressing Ho-1-U-1 cells and control Ho-1-U-1 cells. Data represent the mean ± SD of triplicates in each group. *P-value < 0.05. (**B**) Immunoblotting confirming the expression of TAGLN in TGFBI-overexpressing Ho-1-U-1 cells transfected with TAGLN shRNA. β-actin served as the loading control. (**C**) Assessment of sphere formation using Ultra low attachment surface plates. 1 × 10^4^ cells were seeded into 24-well Ultra low attachment surface plates. Images of spheroid formation are shown after 4 days. (**D**) The number of spheroids with a size of 100 µm or more was counted after 4 days. Data represent the mean ± SD of triplicates in each group. *P-value < 0.05. (**E**) Immunoblotting confirming the expression of HA-tagged TAGLN and TGFBI in TAGLN-overexpressing SAS and OSC20 cells transfected with TGFBI siRNA. β-actin served as the loading control. (**F**) Assessment of sphere formation using Ultra low attachment surface plates. 1 × 10^4^ cells were seeded into 24-well Ultra low attachment surface plates. Images of spheroid formation are shown after 6 days. (**G**) The number of spheroids with a size of 100 µm or more was counted after 6 days. Data represent the mean ± SD in each group (n = 4). *P-value < 0.05. (**H**) TGFBI-induced *TAGLN* expression mediated by FAK. TGFBI-overexpressing Ho-1-U-1 cells showed a decreased expression of *TAGLN*, when treated by FAK inhibitor 14 at indicated concentration. After 3 h treatment, cells were collected and *TAGLN* expression was determined by quantitative RT-PCR. Data represent the mean ± SD of triplicates in each group. *P-value < 0.05. (**I**) The expression of TAGLN was examined by immunoblotting after treatment FAK inhibitor 14 at indicated concentration. β-actin served as the loading control.

Subsequently, we further investigated the involvement of TAGLN in TGFBI-mediated sphere formation. TAGLN shRNA reduced the expression levels of TAGLN mRNA and protein (Fig. 3A,B). As expected, TAGLN depletion suppressed sphere formation (Fig. 3C,D). However, TAGLN depletion did not decrease CSC markers, *ALDH1* and *BCL11B* (Supplementary Figure S7B). Moreover, we examined the impact of TAGLN depletion on sphere formation in TGFBI-overexpressing Ho-1-U-1 cells (Fig. 3A,B). The depletion of TAGLN suppressed sphere formation, as did the sphere formation induced by TGFBI overexpression (Fig. 3C,D). Additionally, we investigated the effect of TAGLN overexpression in TGFBI-depleted SAS and OSC20 cells (Fig. 3E). Interestingly, TAGLN overexpression not only promoted sphere formation on its own but also rescued the sphere formation suppressed by TGFBI depletion (Fig. 3F,G).Given that TGFBI contains an RGD sequence that binds to integrin αvβ3 and αvβ5, with a higher affinity for the αvβ5 complex^12,13^, we explored its potential role in integrin signaling. TGFBI is known as the primary intracellular downstream signaling mediators of integrins and promotes αvβ5 integrin signaling to FAK (focal adhesion kinase), thereby contributing to cancer progression through its integrin-binding RGD motif in osteosarcoma, colon, and pancreatic cancer models^12,14,15^. To investigate this mechanism, we treated TGFBI-overexpressing Ho-1-U-1 cells with the FAK inhibitor 14, which directly inhibits FAK Y397 autophosphorylation. FAK inhibitor downregulated TAGLN expression in a dose-dependent manner (Fig. 3H,I), suggesting that TAGLN expression may be induced through TGFBI-mediated integrin signaling.

### Correlation of TAGLN expression with clinical parameters and TGFBI in HNSCC

To investigate the correlation of *TAGLN* expression with lymph node metastasis and prognosis in HNSCC cases, we analyzed processed RNA-seq data obtained from the TCGA database. High expression of *TAGLN* was associated with advanced grading, lymph node metastasis, and poor prognosis, compared to low expression of *TAGLN* (Supplementary Fig. S8A and B). Although TAGLN expression was dominantly observed in cancer stromal fibroblasts, a certain population of cancer cells also expressed TAGLN (Supplementary Fig. S8C). This result is consistent with the notion that cancer stem cells are a small population within the cancer cells.

**Figure 4:**
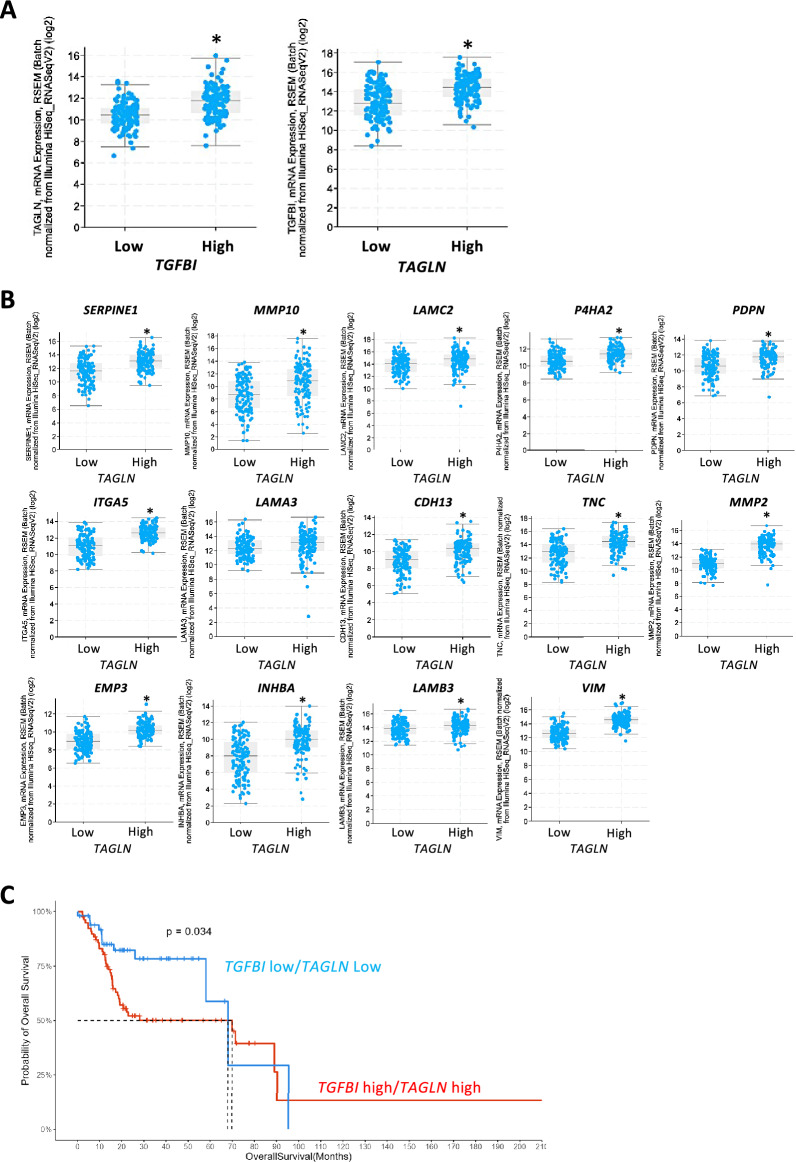
Correlation of *TAGLN* expression with the expression of *TGFBI* and partial-EMT -related genes in HNSCC. (**A**) Correlation between *TAGLN* expression and *TGFBI* expression in HNSCC patients. *P-value < 0.05. (**B**) Correlation between *TAGLN* expression and the expression of partial-EMT-related genes in HNSCC patients. *P-value < 0.05. (**C**) Kaplan–Meier overall survival analysis in HNSCC patients with "low" and "high" *TGFBI*/*TAGLN* expression. HNSCC cases were divided into two groups based on both TGFBI and TAGLN expression levels: "low" (n = 50, bottom 25% patient group) and "high" (n = 78, top 25% patient group).

Additionally, we examined the relationship between TAGLN and TGFBI expression in HNSCC cases, revealing that high expression of TAGLN was correlated with elevated TGFBI expression, and vice versa (Fig. 4A). Moreover, we examined the correlation between TAGLN and partial-EMT-related genes identified in a previous study^8^. Interestingly, HNSCC cases with high TAGLN expression exhibited increased expression of partial-EMT-related genes (Fig. 4B).

Furthermore, we explored the correlation of *TGFBI*/*TAGLN* expression with stem cell markers (*NANOG*, *POU5F1*, *SOX2*, *ALDH1*, and *BCL11B*) and EMT markers (*S100A4* and *VIM*) in HNSCC cells. There was no significant elevation in stem cell markers in HNSCC cases with high expression of *TGFBI* and *TAGLN* compared to those with low expression of these genes (Supplementary Figure S9). However, EMT markers exhibited higher levels in HNSCC cases with high *TGFBI* and *TAGLN* expression (Supplementary Figure S9). We conducted Kaplan–Meier overall survival analysis in HNSCC patients with "low" and "high" *TGFBI*/*TAGLN* expression. Interestingly, HNSCC patients with high *TGFBI*/*TAGLN* expression significantly exhibited an unfavorable prognosis compared to those with low expression of these genes (Fig. 4C).

## Discussion

TGFBI, belonging to the FAS1 family, shares a striking 48% similarity with periostin, a secreted protein renowned for its influential impact^16^. Like TGFBI, periostin contains the EMI and four FAS1 domains, but does not contain an RGD sequence. Our previous study demonstrated that periostin has the ability to promote invasion, angiogenesis, and metastasis in HNSCC^11,17^. TGFBI does not enhance migration and invasion but remarkably promotes sphere formation—an intriguing distinction in its function within HNSCC cells despite their similar protein structures.

Although previous reports have highlighted the dual nature of TGFBI, acting both as a tumor suppressor and a promoter, accumulating evidence underscores its significant effects in driving tumor progression^10^. Single cell transcriptome analysis identifies TGFBI as a marker of partial-EMT^8^. Moreover, bioinformatics analysis pinpoints its identification as a hub gene associated with lymph node metastasis in HNSCC^18^. Also, we previously have shown a significant correlation between TGFBI expression and poor survival among HNSCC patients^19^. Thus, TGFBI acts as a promoter of tumor progression in HNSCC. TGFBI's impact extends beyond mere correlations, encompassing critical aspects such as chemotaxis, migratory potential, proliferation, apoptosis, metastatic niche promotion, cancer cell adhesion, and aberrant angiogenesis in other types of cancer^10,14,15,20–27^. Mechanistically, TGFBI propels cancer progression by activating αvβ5 integrin signaling, ultimately engaging Src, FAK, PI3K, and AKT through its RGD motif, as observed in models of osteosarcoma, colon, and pancreatic cancer^12,14,15^. Indeed, TGFBI is identified as a necessary gene for tumorsphere formation in stem-like breast cancer cells expressing the integrin αvβ3^28^. Although previous study shows that TGFBI-positive HNSCC cells sorted by flow cytometry exhibit increased invasiveness and decreased proliferation^8^, here we demonstrate that TGFBI remarkably enhances the ability of HNSCC cells to form spheres, a hallmark of CSC properties.

In our pursuit of unraveling the molecular mechanisms behind TGFBI-mediated sphere formation, we conducted RNA sequencing analysis, which revealed TAGLN as a gene upregulated in TGFBI-overexpressing cells and highly correlated with TGFBI expression in HNSCC cases from the TCGA database. TAGLN plays pivotal roles in podosome formation, myocyte migration, and vascular and visceral smooth muscle cell differentiation^29^. Although TAGLN is traditionally considered a tumor suppressor in various cancers, our study uncovers elevated TAGLN expression closely associated with adverse clinical parameters, including poor survival, advanced grading, and lymph node metastasis in HNSCC patients. Furthermore, TAGLN expression exhibits a strong correlation with the expression of TGFBI and partial-EMT-related genes, suggesting a crucial role for TAGLN in the aggressiveness and metastatic potential of HNSCC mediated by TGFBI. While the intricate mechanisms governing TAGLN regulation by TGFBI remain elusive, our study hints at the involvement of integrin signaling and its downstream effector, FAK. Notably, the inhibition of FAK downregulates TAGLN expression, suggesting that TGFBI-mediated integrin signaling, facilitated by its RGD motif, may induce TAGLN expression in HNSCC cells. Intriguingly, TGFBI-positive cells exhibiting partial-EMT traits localize at the leading edge of HNSCC tissue^8^, implying potential interactions between TGFBI and the tumor microenvironment, particularly ligand-receptor signaling. Furthermore, TGFBI exhibits enhanced sphere formation with upregulation of CSC markers. However, TAGLN depletion did not contribute to the expression of CSC markers. The correlation between TGFBI-TAGLN axis and stemness is still unknown. The detailed mechanism on the involvement of TGFBI in regulating tumor microenvironment and stemness requires further study.

In conclusion, our study uncovers a TGFBI-TAGLN axis via integrin signaling, underscoring their potential interplay in driving HNSCC. The association of TGFBI and TAGLN with adverse clinical parameters further emphasizes its significance in HNSCC aggressiveness. Our investigations have illuminated the captivating involvement of TGFBI-TAGLN axis in HNSCC, positioning them as promising targets for future therapies.

### Supplementary Information

Supplementary Information 1.

Supplementary Information 2.

## Data Availability

The data supporting the findings of this study are available within the paper and the Supplementary Information. Raw RNA-seq data (FASTQ files) reported in this study are available at the DDBJ Sequence Read Archive (DRR486141-DRR486143).

## References

[1] Mao L, Hong WK, Papadimitrakopoulou VA (2004). Focus on head and neck cancer. Cancer Cell.

[2] Forastiere A, Koch W, Trotti A, Sidransky D (2001). Head and neck cancer. N. Engl. J. Med..

[3] Fidler IJ (1990). Critical factors in the biology of human cancer metastasis: Twenty-eighth GHA Clowes memorial award lec-ture. Cancer Res..

[4] Nieto MA, Huang RY, Jackson RA, Thiery JP (2016). EMT: 2016. Cell.

[5] Lüönd F, Sugiyama N, Bill R, Bornes L, Hager C, Tang F (2021). Distinct contributions of partial and full EMT to breast cancer malignancy. Dev. Cell.

[6] Pal A, Barrett TF, Paolini R, Parikh A, Puram SV (2021). Partial EMT in head and neck cancer biology: A spectrum instead of a switch. Oncogene.

[7] Jolly MK, Tripathi SC, Jia D, Mooney SM, Celiktas M, Hanash SM (2016). Stability of the hybrid epithelial/mesenchymal phenotype. Oncotarget.

[8] Puram SV, Tirosh I, Parikh AS, Patel AP, Yizhak K, Gillespie S (2017). Single-cell transcriptomic analysis of primary and metastatic tumor ecosystems in head and neck cancer. Cell.

[9] Han KE, Choi SI, Kim TI, Maeng YS, Stulting RD, Ji YW (2016). Pathogenesis and treatments of TGFBI corneal dystrophies. Prog. Retin. Eye Res..

[10] Corona A, Blobe GC (2021). The role of the extracellular matrix protein TGFBI in cancer. Cell Signal..

[11] Kudo Y, Ogawa I, Kitajima S, Kitagawa M, Kawai H, Gaffney PM (2006). Periostin promotes invasion and anchorage-independent growth in the metastatic process of head and neck cancer. Cancer Res..

[12] Costanza B, Rademaker G, Tiamiou A, De Tullio P, Leenders J, Blomme A (2019). Transforming growth factor beta-induced, an extracellular matrix interacting protein, enhances glycolysis and promotes pancreatic cancer cell migration. Int. J. Cancer.

[13] Son HN, Nam JO, Kim S, Kim IS (2013). Multiple FAS1 domains and the RGD motif of TGFBI act cooperatively to bind αvβ3 integrin, leading to anti-angiogenic and anti-tumor effects. Biochim. Biophys. Acta..

[14] Guo YS, Zhao R, Ma J, Cui W, Sun Z, Gao B (2014). βig-h3 promotes human osteosarcoma cells metastasis by interacting with integrin α2β1 and activating PI3K signaling pathway. PLoS One.

[15] Ma C, Rong Y, Radiloff DR, Datto MB, Centeno B, Bao S (2008). Extracellular matrix protein betaig-h3/TGFBI promotes metastasis of colon cancer by enhancing cell extravasation. Genes Dev..

[16] Skonier J, Neubauer M, Madisen L, Bennett K, Plowman GD, Purchio AF (1992). cDNA cloning and sequence analysis of βig-h3, a novel gene induced in a human adenocarcinoma cell line after treatment with transforming growth factor-β. DNA Cell Biol..

[17] Siriwardena BS, Kudo Y, Ogawa I, Kitagawa M, Kitajima S, Hatano H (2006). Periostin is frequently overexpressed and enhances invasion and angiogenesis in oral cancer. Br. J. Cancer.

[18] Lu H, Li L, Sun D, Duan Y, Yue K, Wu Y (2021). Identification of novel hub genes associated with lymph node metastasis of head and neck squamous cell carcinoma by completive bioinformatics analysis. Ann. Transl. Med..

[19] Kisoda S, Shao W, Fujiwara N, Mouri Y, Tsunematsu T, Jin S (2020). Prognostic value of partial EMT-related genes in head and neck squamous cell carcinoma by a bioinformatic analysis. Oral Dis..

[20] Lee MJ, Heo SC, Shin SH, Kwon YW, Do EK, Suh DS (2013). Oncostatin M promotes mesenchymal stem cell-stimulated tumor growth through a paracrine mechanism involving periostin and TGFBI. Int. J. Biochem. Cell Biol..

[21] Shang D, Song B, Liu Y (2018). Epirubicin suppresses proliferative and metastatic potential by downregulating transforming growth factor-β-induced expression in urothelial carcinoma. Cancer Sci..

[22] Shin SH, Kim J, Heo SC, Kwon YW, Kim YM, Kim IS (2021). Proteomic identification of betaig-h3 as a lysophosphatidic acid induced secreted protein of human mesenchymal stem cells: Paracrine activation of A549 lung adenocarcinoma cells by betaig-h3. Mol. Cell Proteomics..

[23] Guo SK, Shen MF, Yao HW, Liu YS (2018). Enhanced expression of TGFBI promotes the proliferation and migration of glioma cells. Cell Physiol. Biochem..

[24] Shang D, Liu Y, Yang P, Chen Y, Tian Y (2021). TGFBI-promoted adhesion, migration and invasion of human renal cell carcinoma depends on inactivation of von Hippel-Lindau tumor suppressor. Urology.

[25] Pan T, Lin SC, Yu KJ, Yu G, Song JH, Lewis VO (2018). BIGH3 promotes osteolytic lesions in renal cell carcinoma bone metastasis by inhibiting osteoblast differentiation. Neoplasia.

[26] Steitz AM, Steffes A, Finkernagel F, Unger A, Sommerfeld L, Jansen JM (2020). Tumor-associated macrophages promote ovarian cancer cell migration by secreting transforming growth factor beta induced (TGFBI) and tenascin C. Cell Death Dis..

[27] Chiavarina B, Costanza B, Ronca R, Blomme A, Rezzola S, Chiodelli P (2021). Metastatic colorectal cancer cells maintain the TGFβ program and use TGFBI to fuel angiogenesis. Theranostics.

[28] Sun Q, Wang Y, Officer A, Pecknold B, Lee G, Harismendy O (2022). Stem-like breast cancer cells in the activated state resist genetic stress via TGFBI-ZEB1. NPJ Breast Cancer.

[29] Thweatt R, Lumpkin CK, Goldstein S (1992). A novel gene encoding a smooth muscle protein is overexpressed in senescent human fibroblasts. Biochem. Biophys. Res. Commun..

