# Supplementary material for: Involvement of TGFBI-TAGLN axis in cancer stem cell property of head and neck squamous cell carcinoma
Source: Sci Rep. 2024 Mar 21;14:6767. doi: 10.1038/s41598-024-57478-0 (PMC10957997; doi:10.1038/s41598-024-57478-0)
Supplement: Supplementary file 2 — Supplementary Information 2. [file 41598_2024_57478_MOESM2_ESM.pdf]

Supplementary Figure S1

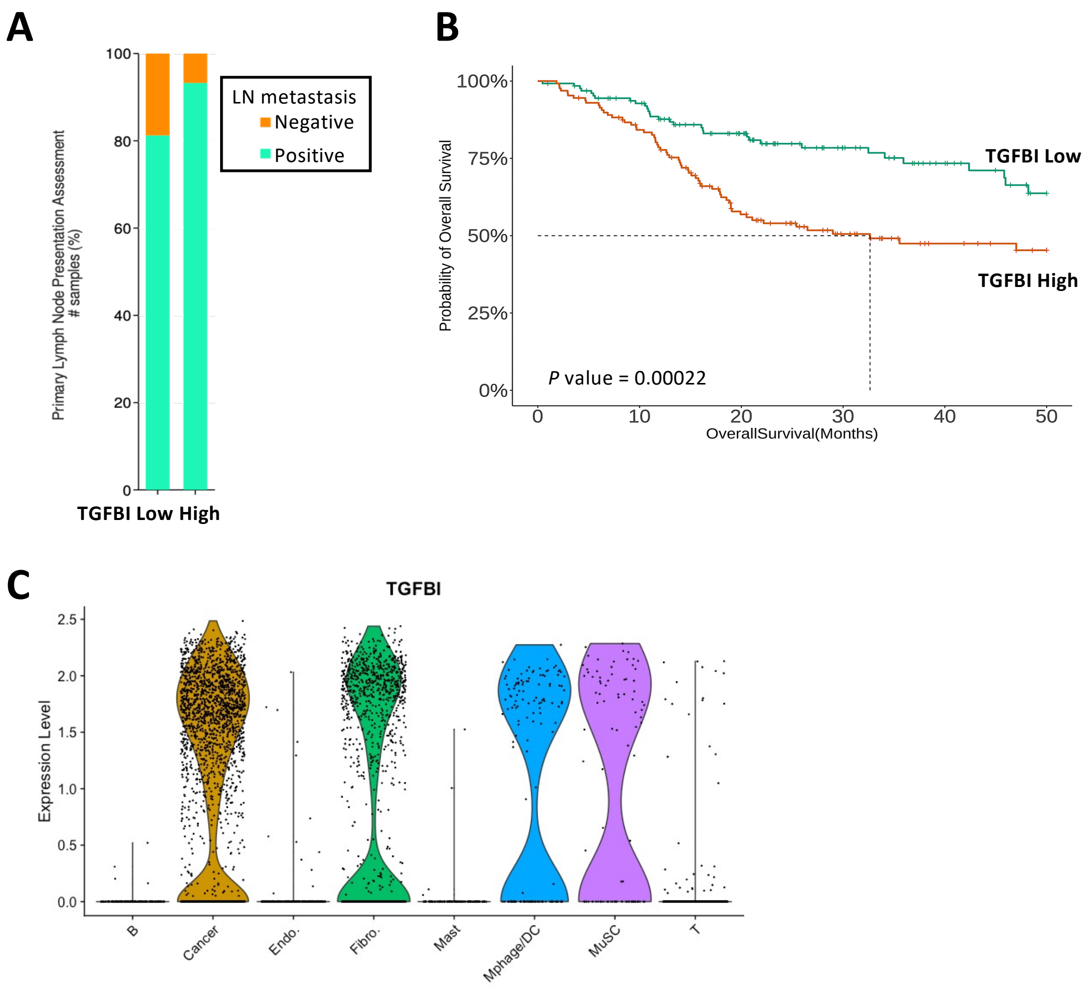

Supplementary Figure S1. *Malignant behaviors and expression patterns of TGFBI in HNSCC.* (A) Assessment of primary lymph node presentation in HNSCC patients based on *TGFBI* expression levels. TCGA-HNSCC RNA-seq data and clinical information were utilized to divide HNSCC cases into two groups: "low" (n=128, bottom 25% patient group) and "high" (n=128, top 25% patient group) *TGFBI* expression. (B) Kaplan-Meier overall survival analysis in HNSCC patients with "low" and "high" *TGFBI* expression. (C) The violin plot shows the *TGFBI* expression in each cell type from HNSCC scRNA-seq data (GSE103322).

Supplementary Figure S2

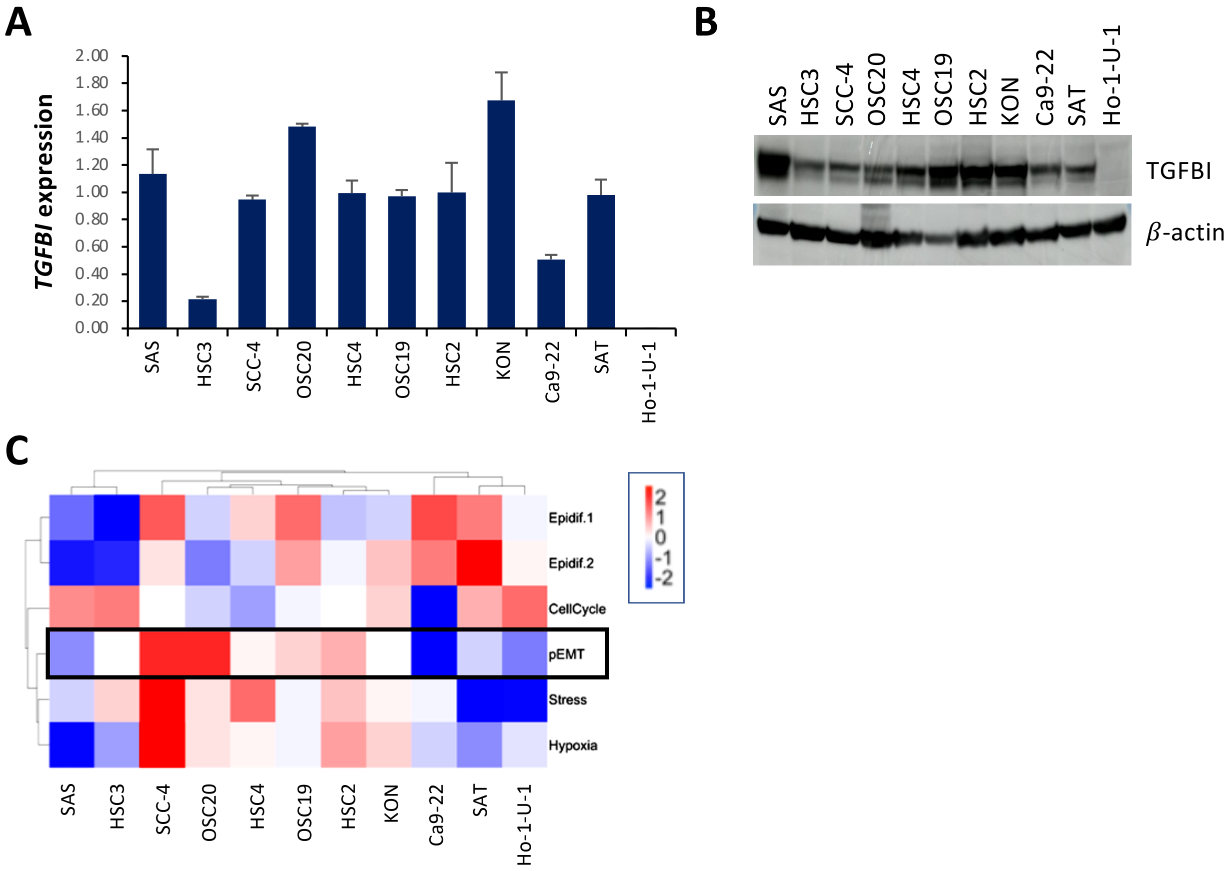

Supplementary Figure S2. *Correlation of TGFBI expression with partial-EMT phenotype in HNSCC cell lines.* (A) Quantitative RT-PCR analysis of *TGFBI* expression in HNSCC cell lines. Data represent the mean ± SD of technical triplicates. (B) Expression of TGFBI was examined in HNSCC cell lines by immunoblotting. β-actin was used as a loading control. (C) Heat map displaying the scores of six gene programs (CellCycle, Epidif.1/2, Stress, Hypoxia, and pEMT) in various HNSCC cell lines.

Supplementary Figure S3

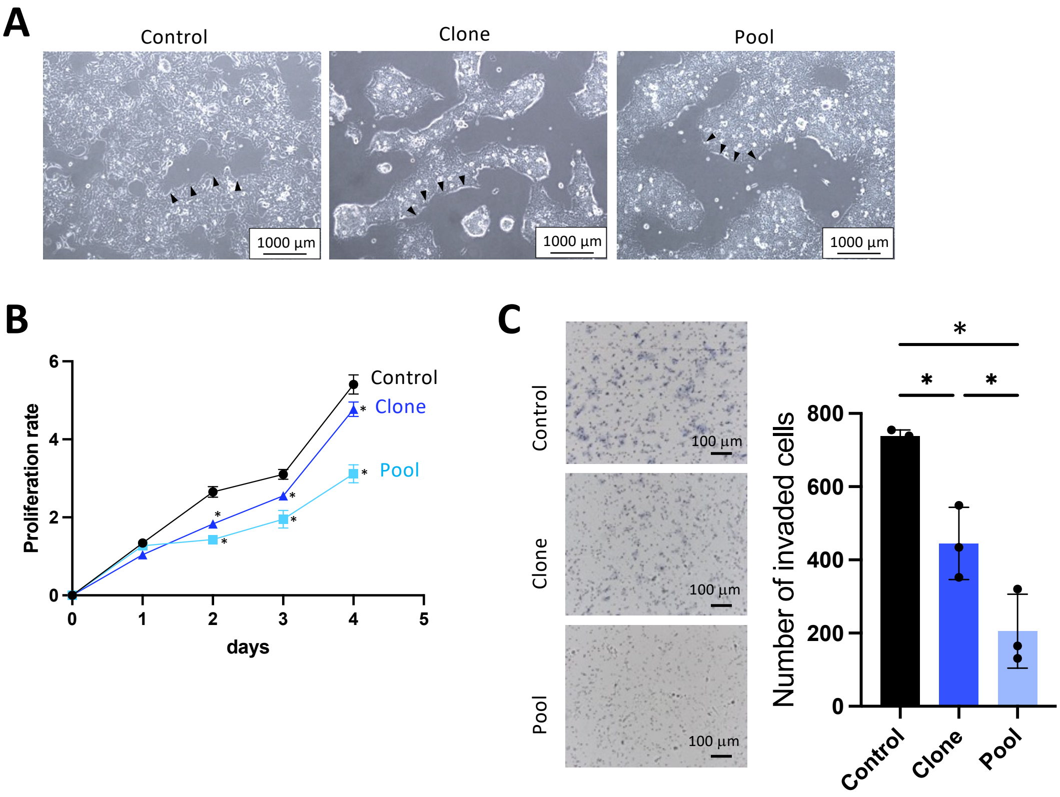

Supplementary Figure S3. *Suppression of cell proliferation and invasion by TGFBI overexpression.* (A) Morphology of TGFBI-overexpressing Ho-1-U-1 cells. TGFBI-overexpressing Ho-1-U-1 cells showed smooth margin shapes (arrow heads), compared with control cells. (B) Proliferation assay of TGFBI-overexpressing Ho-1-U-1 cells (n=3). *P value < 0.001. (C) Invasion assay of TGFBI-overexpressing Ho-1-U-1 cells (n=3). *P value < 0.05.

Supplementary Figure S4

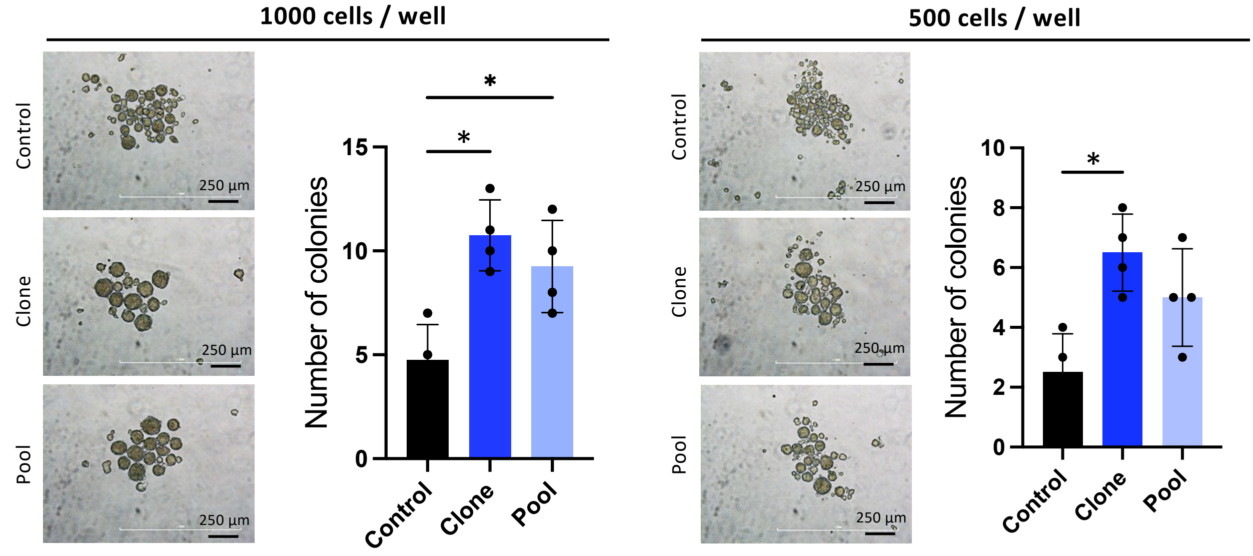

Supplementary Figure S4. *Sphere formation induced by TGFBI.* Enhanced sphere formation in low number of cells by TGFBI overexpression. Sphere formation assay of TGFB-overexpressing Ho-1-U-1 cells in low number of cells (1000 cells/well, 500 cells/well). Sphere formation was assessed by using Ultra low attachment surface plates. $1x10^4$ cells were seeded into 24-well Ultra low attachment surface plates. After 4 days, the number of spheroids with a size of 100 µm or more was counted. Data are presented as the mean ± SD in each group (n=4). *P-value < 0.05.

Supplementary Figure S5

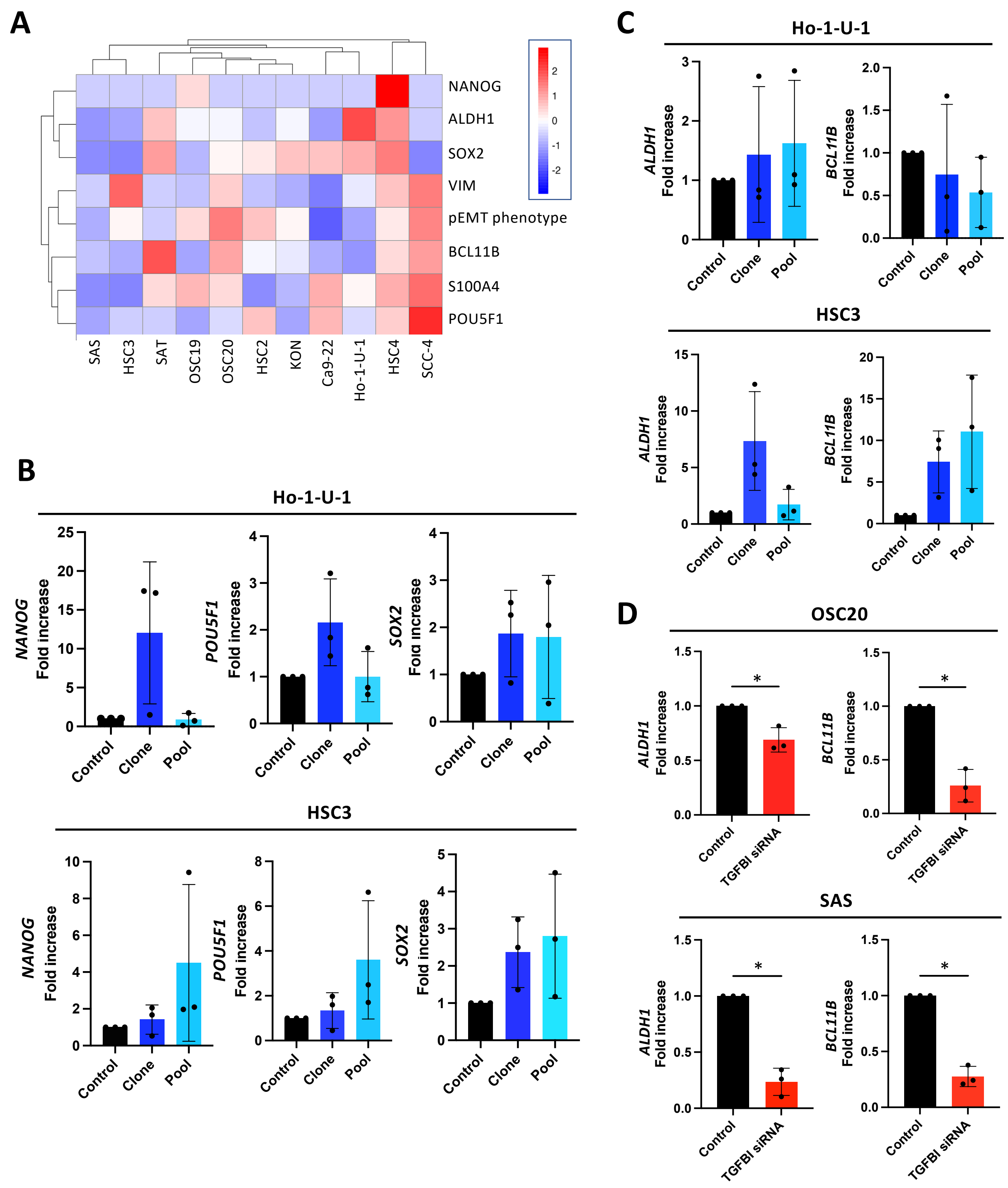

Supplementary Figure S5. *Correlation between TGFBI and CSC markers.* (A) Heat map displaying the expression of stem cell markers (*NANOG*, *POU5F1*, *SOX2*, *ALDH1*, and *BCL11B*) and EMT markers (*VIM* and *S100A4*) in various HNSCC cell lines. (B) Expression of stem cell markers by TGFBI overexpression. Quantitative RT-PCR analysis of *Nanog*, *OCT4*, and *SOX2* expression in TGFBI-overexpressing Ho-1-U-1 and HSC3 cells (clone and pool) and control cells. Data represent the mean ± SD of triplicates in each group. (C) Expression of CSC markers expression by TGFBI overexpression. Quantitative RT-PCR analysis of *ALDH1* and *BCL11B* expression in TGFBI-overexpressing Ho-1-U-1 and HSC3 cells (clone and pool) and control cells. Data represent the mean ± SD of triplicates in each group. (D) Downregulation of CSC markers by TGFBI depletion. Quantitative RT-PCR analysis of CSC markers *ALDH1* and *BCL11B* expression in control and TGFBI-knockdown OSC20 and SAS cells. Data represent the mean ± SD of triplicates in each group. **P*-value<0.05.

Supplementary Figure S6

Upregulated genes

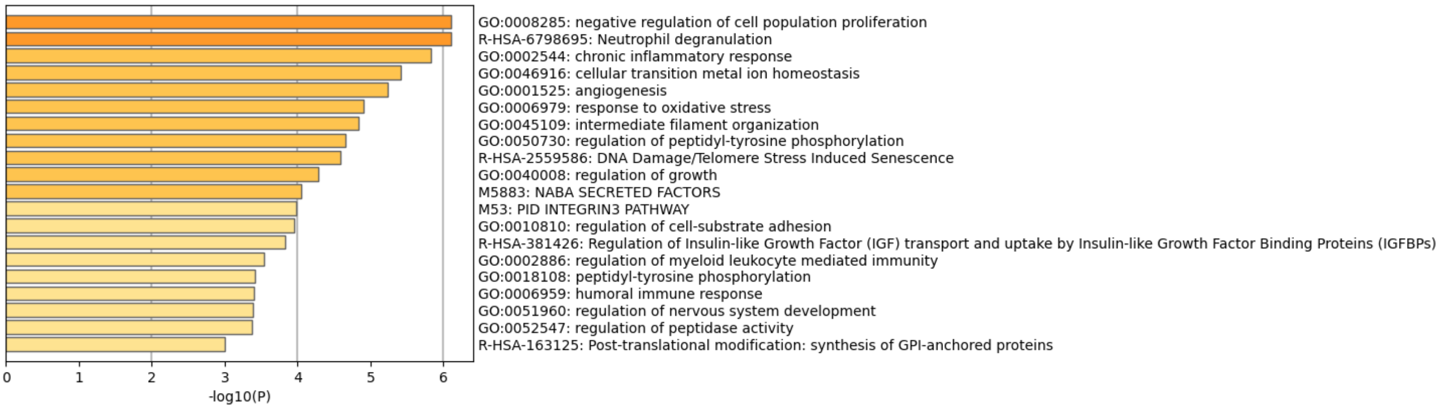

Downregulated gene

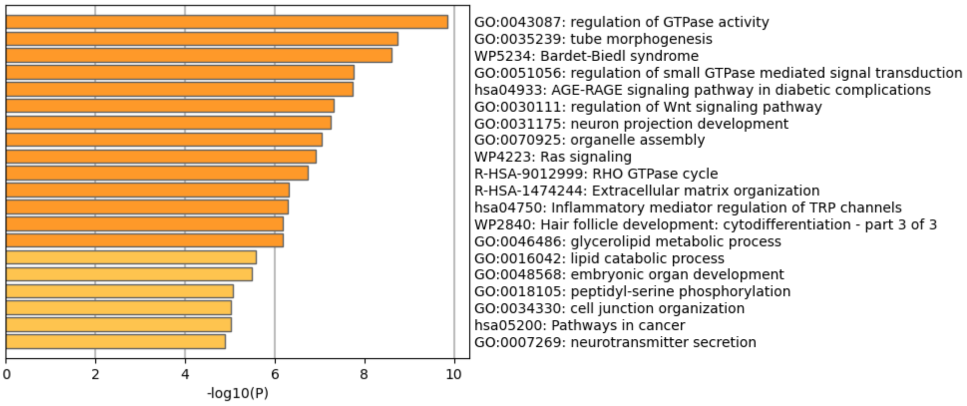

Supplementary Figure S6. *GO gene enrichment of TGFBI-overexpressing cells.* GO gene enrichment analysis shows TGFBI related gene sets. Metascape web-based tool (http://metascape.org/gp/index.html#/main/step1) and Ho-1-U-1 TGFBI overexpressing clone cell's RNA-seq data were used for analysis.

Supplementary Figure S7

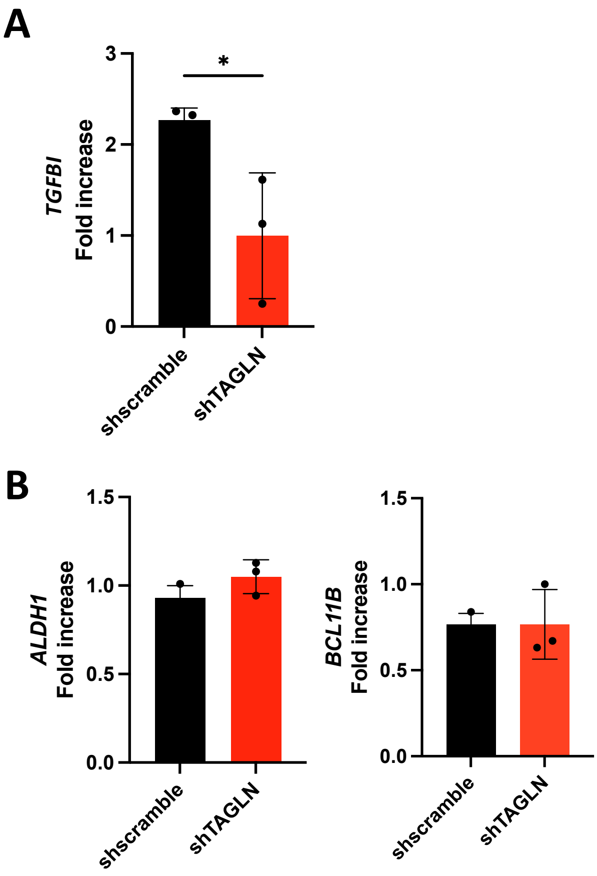

Supplementary Figure S7. *Expression of TGFBI and CSC markers by TAGLN depletion.* (A) Quantitative RT-PCR analysis of *TGFBI* expression in control (shscramble) and TAGLN shRNA in Ho-1-U-1 cells. Data represent the mean ± SD of triplicates in each group. *P<0.05. (B) Quantitative RT-PCR analysis of CSC markers, *ALDH1* and *BCL11B* expression in control (shscramble) and TAGLN shRNA in Ho-1-U-1 cells. Data represent the mean ± SD of triplicates in each group.

Supplementary Figure S8

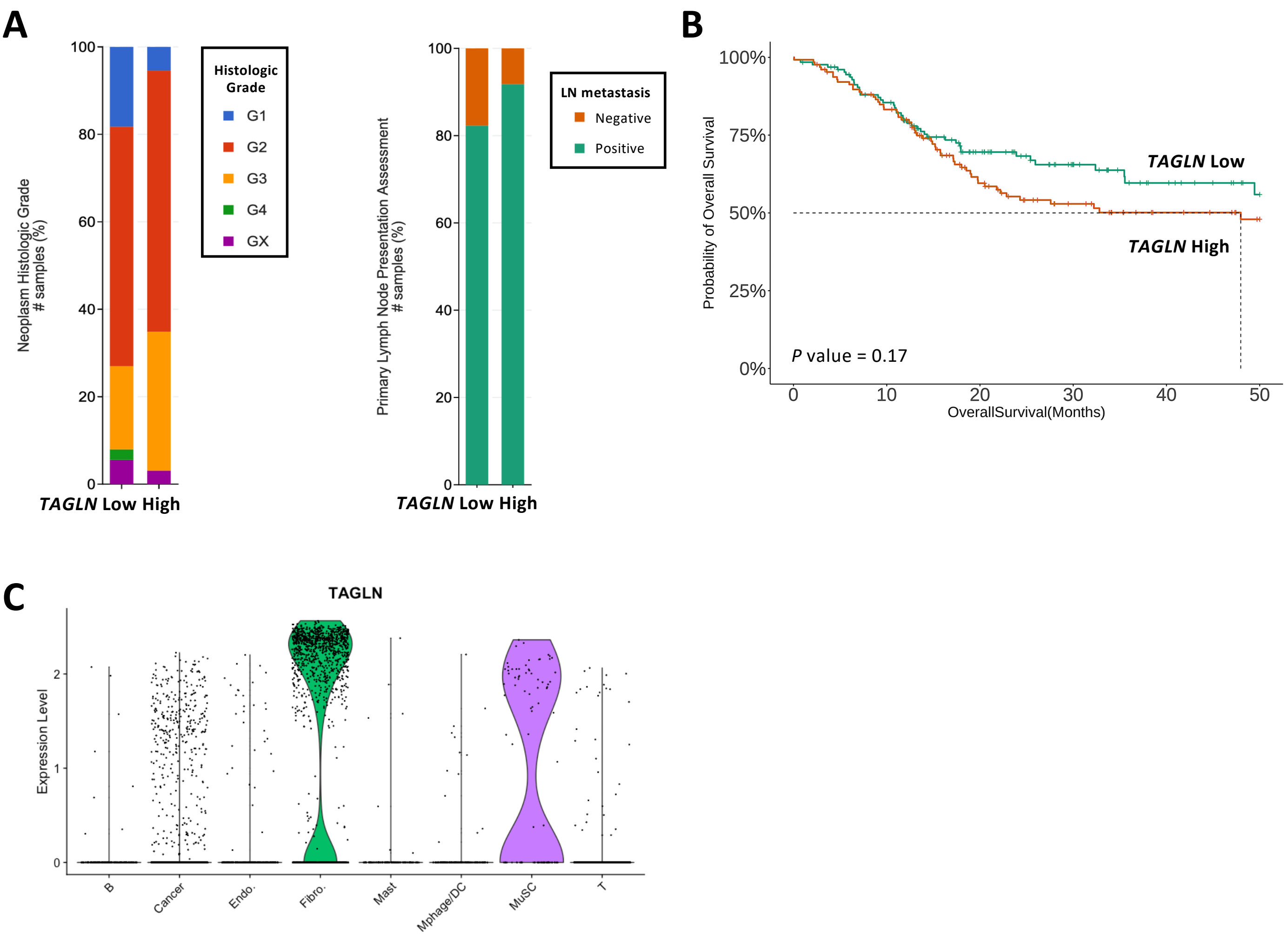

Supplementary Figure S8. *Correlation of TAGLN expression with clinical parameters in HNSCC.* (A) Assessment of primary lymph node presentation in HNSCC patients with "low" and "high" *TAGLN* expression using data from TCGA PanCancer atlas. HNSCC cases were divided into two groups based on *TAGLN* expression levels: "low" (n=128, bottom 25% patient group) and "high" (n=128, top 25% patient group). (B) Kaplan-Meier overall survival analysis in HNSCC patients with "low" and "high" *TAGLN* expression. (C) The violin plot shows the *TAGLN* expression in each cell type from HNSCC scRNA-seq data (GSE103322).

Supplementary Figure S9

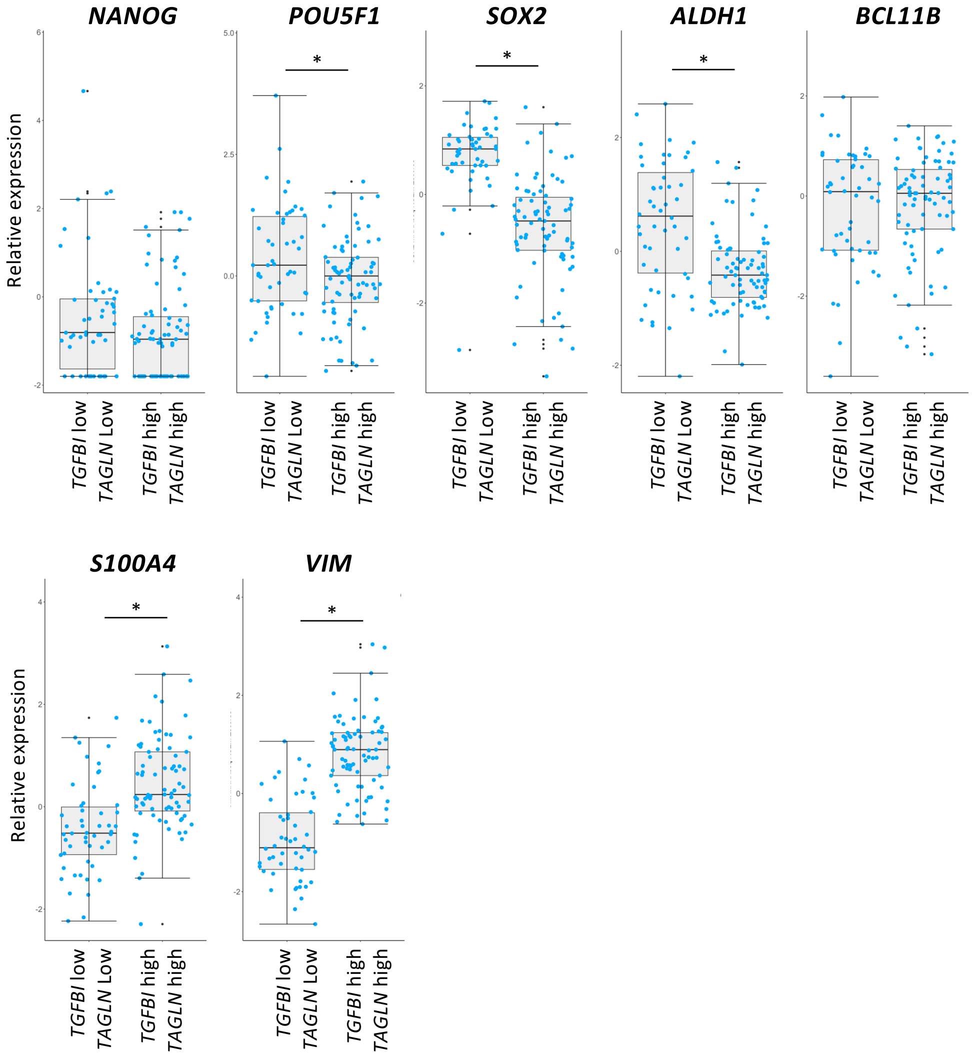

Supplementary Figure S9. *Correlation of TGFBI and TAGLN expression with stem cell and EMT markers in HNSCC.* Correlation of "low" and "high" TGFBI/TAGLN expression with the expression of stem cell markers (*NANOG*, *POU5F1, SOX2*, *ALDH1*, and *BCL11B*) and EMT markers (*S100A4* and *VIM*) in HNSCC patients was examined using data from TCGA PanCancer atlas. HNSCC cases were divided into two groups based on both *TGFBI* and *TAGLN* expression levels: "low" (n=50, bottom 25% patient group) and "high" (n=79, top 25% patient group).

Supplementary Figure S10

A. Corresponds to the panel Figure 1A

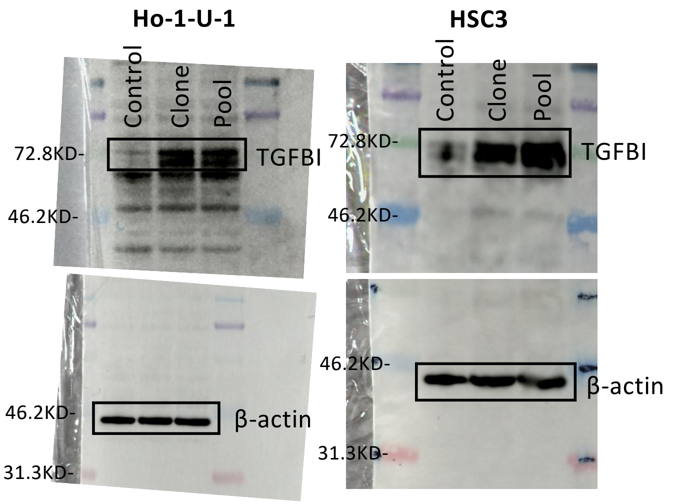

B. Corresponds to the panel Figure 3B

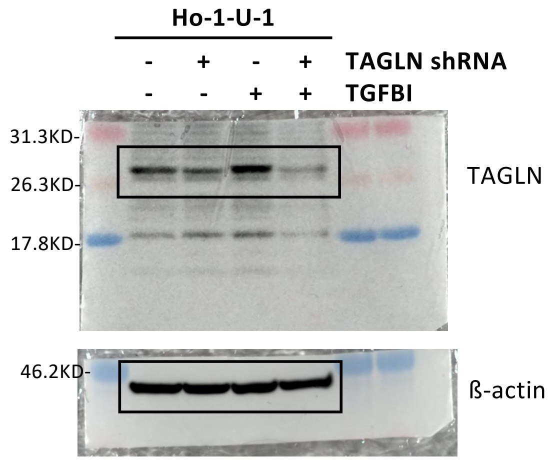

C. Corresponds to the panel Figure 3E

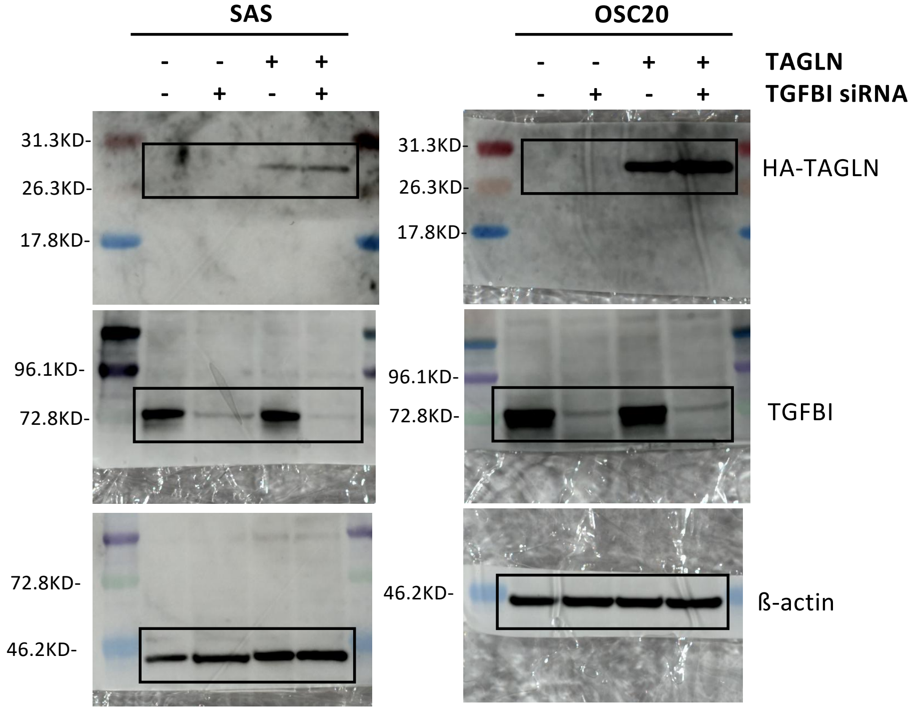

D. Corresponds to the panel Figure 3I

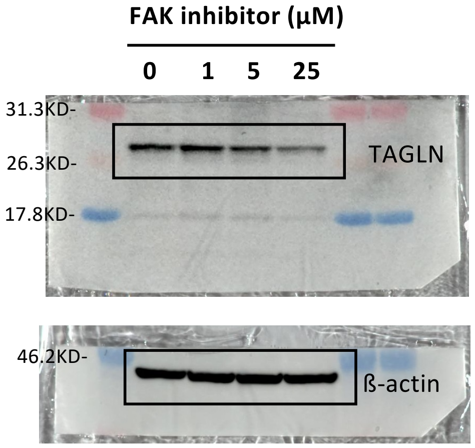

E. Corresponds to the panel Supplementary Figure S2B

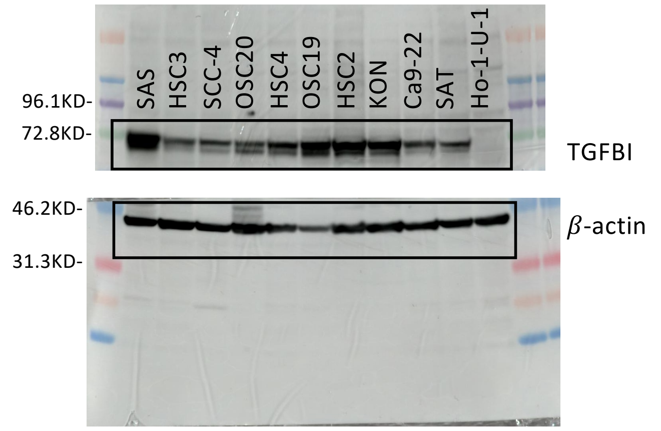

Supplementary Figure S10. *Unprocessed original scans of Western blot analysis.* Each panel indicates the figure to which the full membranes belong (A–E). Molecular mass size standards are indicated on each membrane.
